# Evaluation of native and non‐native biomaterials for engineering human skin tissue

**DOI:** 10.1002/btm2.10297

**Published:** 2022-02-21

**Authors:** Carolina Motter Catarino, Katharina Kaiser, Tânia Baltazar, Luiza Motter Catarino, Jonathan R. Brewer, Pankaj Karande

**Affiliations:** ^1^ Howard P. Isermann Department of Chemical and Biological Engineering Rensselaer Polytechnic Institute Troy New York USA; ^2^ Center for Biotechnology and Interdisciplinary Studies Rensselaer Polytechnic Institute Troy New York USA; ^3^ Department of Biochemistry and Molecular Biology University of Southern Denmark Odense Denmark; ^4^ Department of Biomedicine Positivo University Curitiba Brazil; ^5^ Present address: Department of Immunobiology Yale School of Medicine New Haven Connecticut USA

**Keywords:** 3D skin models, in vitro models, skin grafts, tissue engineering

## Abstract

A variety of human skin models have been developed for applications in regenerative medicine and efficacy studies. Typically, these employ matrix molecules that are derived from non‐human sources along with human cells. Key limitations of such models include a lack of cellular and tissue microenvironment that is representative of human physiology for efficacy studies, as well as the potential for adverse immune responses to animal products for regenerative medicine applications. The use of recombinant extracellular matrix proteins to fabricate tissues can overcome these limitations. We evaluated animal‐ and non‐animal‐derived scaffold proteins and glycosaminoglycans for the design of biomaterials for skin reconstruction in vitro. Screening of proteins from the dermal‐epidermal junction (collagen IV, laminin 5, and fibronectin) demonstrated that certain protein combinations when used as substrates increase the proliferation and migration of keratinocytes compared to the control (no protein). In the investigation of the effect of components from the dermal layer (collagen types I and III, elastin, hyaluronic acid, and dermatan sulfate), the primary influence on the viability of fibroblasts was attributed to the source of type I collagen (rat tail, human, or bovine) used as scaffold. Furthermore, incorporation of dermatan sulfate in the dermal layer led to a reduction in the contraction of tissues compared to the control where the dermal scaffold was composed primarily of collagen type I. This work highlights the influence of the composition of biomaterials on the development of complex reconstructed skin models that are suitable for clinical translation and in vitro safety assessment.

## 1 INTRODUCTION

Tissue engineering is a rapidly advancing multidisciplinary research field with significant promise for regenerative medicine that aims to fabricate and develop tissues and organs in vitro to replace those damaged by disease or injury.^1^, ^2^ For example, bioengineered skin grafts could be used to treat cutaneous injuries, burns as well as chronic and deep wounds.^1^ In tissue engineering, cells are seeded with, or within, a biocompatible matrix or scaffold, which provides the structural integrity as well as the physiological/biochemical cues for cells.^1^, ^3^ Ideally, over time, the cells start producing their own extracellular matrix (ECM), thereby remodeling the tissue. Beyond the applications for regenerative medicine, these tissues and tridimensional models of human organs have been explored as in vitro platforms for efficacy and toxicological screening of substances.^3^ The engineered skin models are typically formed by fibroblasts embedded in a matrix of type I collagen to mimic the dermal layer, and layered with keratinocytes which provide a precursor for the epidermis. A combination of the optimal physiological conditions, such as media composition and exposure to air‐liquid interface, promotes differentiation of the keratinocytes resulting in a stratified epidermal layer. These models have been used for a long time for efficacy and safety assessment of substances as well as start pointing for the study and development of skin grafts for regenerative medicine. Nonetheless, despite the many similarities of these structures with human skin, they still fail in recapitulating the entire complexity of the native tissue.^4^

In vivo, the skin is formed by several types of cells, and comprises a rich combination of molecules such as fibrous proteins, proteoglycans, glycoproteins, soluble growth factors, and signaling molecules all of which are precisely arranged within each skin layer and skin appendages promoting proliferation, migration, and differentiation of constituent cells.^5^, ^6^, ^7^ For example, the dermal matrix is composed mainly of collagen types I, III and V, representing, respectively, 85–90%, 8–11%, and 2–4% of the total collagen content of the dermis in adults.^6^, ^8^ Collagen I provides support and biochemicals cues to the cells in the dermal compartment and epidermal region while type III collagen is involved in the formation of the reticular skin.^9^, ^10^ Interestingly, an increase in the ratio of collagen I/III has been associated with an increase in scar formation during wound healing.^10^ Other important molecules, such as the proteoglycans perlecan, hyaluronic acid and dermatan sulfate, as well as the glycoproteins fibronectin and elastin, are also present at the dermal layer.^6^, ^11^ In skin, elastin is found at a ratio of 1:9 to collagen and is responsible for the elastic properties of the tissue.^12^, ^13^ Hyaluronic acid is a natural glycosaminoglycan that has been associated with tissue homeostasis, angiogenesis, and wound healing.^14^ It is a biocompatible material, with low adhesive properties and extracellular matrix hydration properties, that can be produced by recombinant technology, making it an attractive alternative to animal derived matrices that can pose a risk of immune activation in grafts intended for transplantation to address wound healing.^9^, ^14^ In wound healing, hyaluronic acid has also been associated with reduction of scar formation due to its highly hydrophilic characteristics.^15^ Dermatan sulfate represents 0.3–1% of the dry weight of skin and has been associated with the processes of coagulation, cell growth, immune defense and wound repair.^16^, ^17^ In skin, dermatan sulfate is covalently bound to proteins and performs important functions such as water retention, filling up void spaces, and interacting with cytokines and cell receptors.^18^

At the junction between the dermal and epidermal compartments, several biomolecules, such as proteoglycans, collagen type IV and glycoproteins (laminin 5 and fibronectin), form the basement membrane. In addition to ensuring the adhesion between the two compartments, this thin bilayer membrane regulates the differentiation and proliferation of epidermal cells and is an important reservoir of cytokines and growth factors.^19^, ^20^ Furthermore, the basement membrane is a key component for adhesion of melanocytes and melanin production which directly influences skin pigmentation.^4^

Collagen IV is an adhesive protein of the basement membrane that regulates keratinocyte attachment, proliferation, and differentiation in vivo and *in vitro*.^21^, ^22^ Laminin 5 is an adhesive protein formed by three chains: α, β, and γ.^23^ It promotes adhesion of keratinocytes to the dermal layer and is a known key component of keratinocyte migration in epidermal wound healing (reviewed by Varkey et al.).^20^ Fibronectin is also connected to enhanced cell attachment and migration during re‐epithelization process in wound healing (reviewed by Jahoda et al.).^24^ In cutaneous wounds, the provisional matrix in the damaged region is enriched with fibronectin and fibril‐like proteins produced by fibroblasts that migrate from the subdermal tissue.^25^, ^26^

Biomaterials currently employed for engineering skin models are typically collagen‐based with chemical/temperature‐controlled crosslinking mechanisms. The lack of complexity as well as the diversity of biomolecules and cells found in the native tissue contribute, for example, to the poor mechanical properties characteristics of most in vitro models.^27^ Different approaches have been explored to successfully produce stable dermal layer such as chemical crosslinking associated with lyophilization of scaffold, use of non‐woven hyaluronic acid‐based fibrous material, highly porous polystyrene scaffold among others, but these still rely on a single or a minimum number of components.^28^, ^29^, ^30^, ^31^, ^32^Ralston et al. showed that human acellular dermis separated from the epidermis but retaining the dermal epidermal junction (DEJ) basement membrane induced a significantly higher production of soluble fibronectin by the epidermal cells in in vitro models compared to the control (lack of the basement membrane antigens).^33^ They further demonstrated that the presence of this layer in the DEJ model improved attachment and morphological aspects of the epithelial cells and exhibited the presence of consistent amounts of collagen IV and laminin 5 at the basement membrane as well as increased expression of soluble collagen IV and fibronectin compared to control.^34^ These results suggest that the incorporation of basement membrane proteins within the DEJ can promote not only cell attachment, migration and differentiation, but also the expression of extra cellular matrix proteins, supporting tissue remodeling. Work by El Ghalbzouri et al. demonstrated that the source, that is, human‐ versus animal‐derived, and composition of biomaterials used to generate the skin model have significant influence also on the time over which these tissues can be cultured in vitro.^35^ This is particularly important because the short life span of most current in vitro skin models limits their use in long‐term efficacy or toxicity studies.^35^ They further demonstrated that matrices derived from human primary fibroblasts can support the culture of in vitro models, with a live layer of human primary epithelial cells in the epidermis, for a longer period of time compared to models employing animal‐derived matrices such as rat tail collagen.^35^ In these studies, the model generated with animal‐derived matrix presented a higher degree of tissue contraction compared to a human‐derived matrix, which resulted in poor epidermal homeostasis. The investigators speculate that the lack of a human‐derived matrix could be attenuating the self‐renewal process of keratinocytes.^35^ These characteristics present in in vitro models developed with animal derived matrices can be associated with their short life span. Recently, Kim et al. showed that a complex dermal hydrogel composed of a porcine skin‐derived extracellular matrix, which retained the major biomolecular composition of skin, was able to support its regeneration in vitro with reduced contraction, improved barrier properties and epidermal organization compared to the control (a collagen I‐based hydrogel).^27^ Furthermore, they demonstrated that the porcine skin‐derived extracellular matrix generated a skin construct with a complex moduli 10 times greater than the control sample generated with only collagen I, indicating the importance of the synergistic physical and chemical interaction between the different extracellular matrix components of the skin.^27^ Researchers have also used porous scaffolds to support production of de novo ECM by skin cells, contributing to improved biomolecular complexity, mechanical properties and reproducibility of the skin model.^32^, ^36^, ^37^

A comprehensive effort to develop defined compositions that could sufficiently mimic the inter‐ and intra‐complexity of the various compartments of human skin is currently lacking. This creates an opportunity for the formulation of materials for the development of skin models with increased complexity and defined composition aiming at the generation of reconstructed human skin models that better mimic the native tissue. These efforts should focus not only on increasing the complexity of current skin models in terms of structures, cells, and composition but also in terms of their mechanical properties, host resorbability and integration (relevant for regenerative medicine) and in vitro life span (relevant for efficacy testing).^38^ In this work, we aim to address some of these challenges by investigating the effect of incorporating a broader and expanded repertoire of native biomolecules within the dermal layer and at the dermal‐epidermal junction. We hope that this work will encourage the development of complex reconstructed skin models by including biomaterial combinations that could support the incorporation of more cell types.

## 2 RESULTS

### 2.1 Evaluation of basement membrane proteins for the reconstruction of the dermal‐epidermal junction of skin

#### 2.1.1 Influence of the source of primary cells and collagen coating on proliferation rate of keratinocytes

**FIGURE 1:**
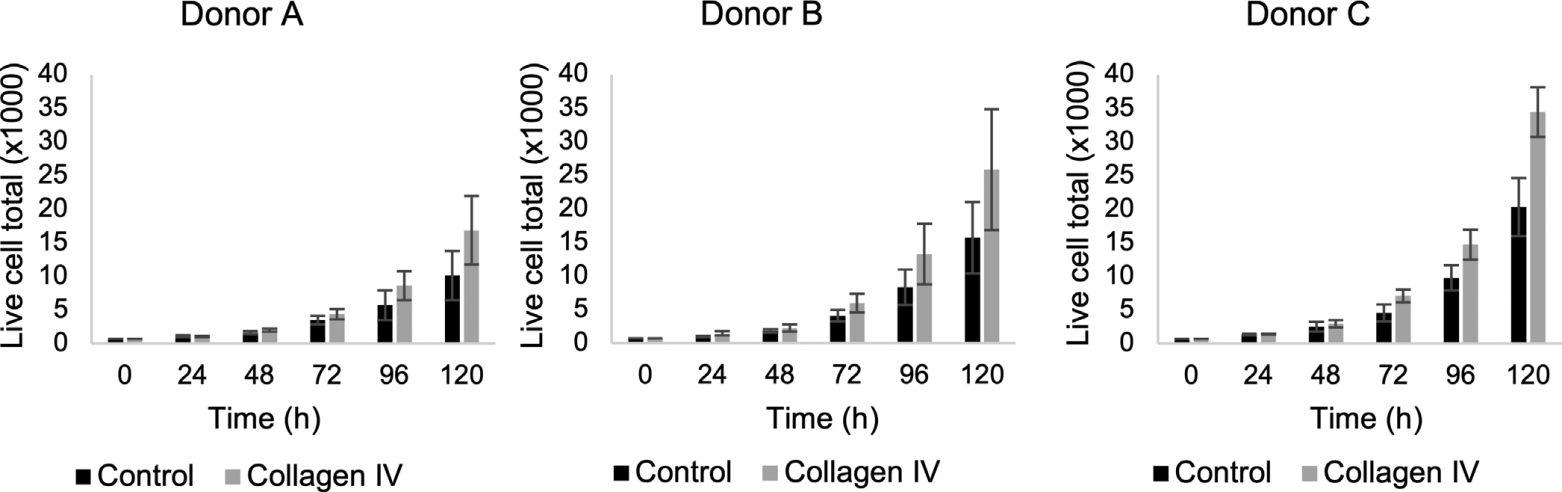
Effect of collagen IV coating (2 μg/cm^2^) on the proliferation rate of keratinocytes. Keratinocytes were isolated from three independent donors: A (female adult breast skin), B (neonatal foreskin), and C (neonatal foreskin). Results present the average ± SD from two independent experiments each performed in triplicate (*n* = 6)

We tested primary human keratinocytes isolated from discarded skin tissue obtained from three independent and de‐identified donors: donor A (adult female, breast, phototype I‐II), B (neonatal male, foreskin, phototype I‐II), and C (neonatal male, foreskin, phototype V‐VI). Figure 1 shows the proliferation rates of cells from each donor in culture on collagen IV (2 μg/cm^2^) coated and uncoated substrates. The proliferation rate was dependent on the age of the donor (adult vs. neonatal), and, as expected, cells isolated from neonatal foreskin (donors B and C) exhibited a higher proliferation rate compared to those isolated from adult breast skin. It can also be observed that cells seeded on substrate coated with collagen (specifically, Type IV) proliferated faster compared to those seeded on an uncoated substrate.

#### 2.1.2 High‐throughput screening of proteins constituting the dermal‐epidermal junction

**FIGURE 2:**
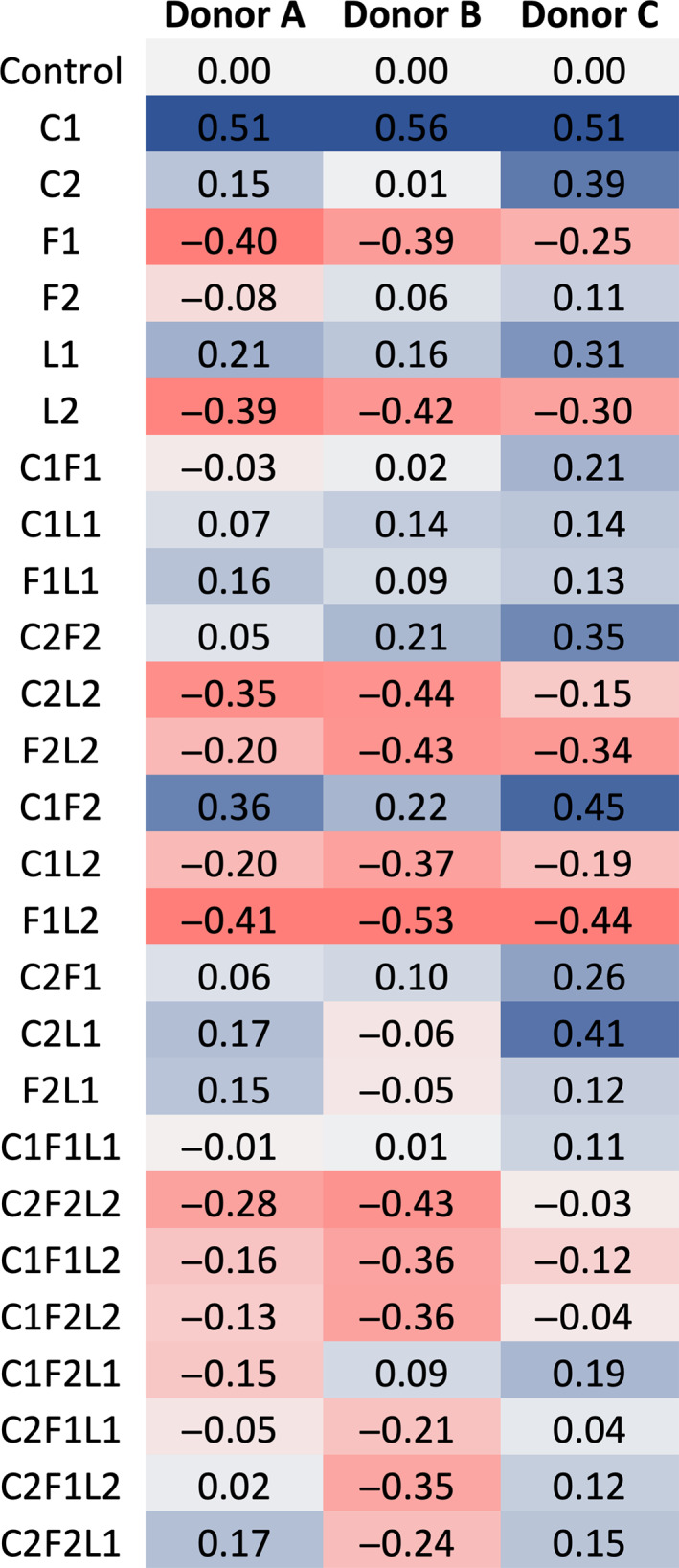
Heat map representing the effect of basement membrane proteins at the dermal‐epidermal junction on proliferation of keratinocytes obtained from donors A, B, and C at Day 4. The color scale represents scoring of the combinations. Blue (corresponding to rate of proliferation > control [uncoated substrate]) represents the highest proliferation rate and Red (corresponding to rate of proliferation < control) represents the lowest proliferation rate of keratinocytes for each donor. Protein concentrations tested: Collagen IV (C)—2 μg/cm^2^ (C1) and 8 μg/cm^2^ (C2), fibronectin (F)—1 μg/cm^2^ (F1), and 4 μg/cm^2^ (F2), and laminin 5 (L)—0.5 μg/cm^2^ (L1) and 2 μg/cm^2^ (L2). Proteins and their combinations were tested in two independent sets of experiments each performed in triplicate (*n* = 6)

Individual, binary, and ternary combinations of collagen IV, fibronectin, and laminin 5 were formulated to mimic the composition and ability of the basement membrane to promote proliferation of keratinocytes and evaluated as described in Materials and Methods. Figure 2 shows a heat map of the various combinations according to their effect on the proliferation rate of keratinocytes (red scale: low proliferation; blue scale: high proliferation) for each of the three individual donors. It can be observed that some of the conditions tested, for example, coating with collagen IV at 2 μg/cm^2^, C1, led to an increase in the rate of proliferation of keratinocytes. For several other conditions, for example, coating with fibronectin at 1 μg/cm^2^ (F1), the proliferation rate of cells was lower compared to the control (uncoated tissue culture‐treated substrate). The same general trend regarding promotion/inhibition of proliferation was observed for cells from the three donors across most of the combinations tested.

#### 2.1.3 Time‐lapse video microscopy of keratinocytes seeded on surfaces coated with proteins constituting the dermal‐epidermal junction

**FIGURE 3:**
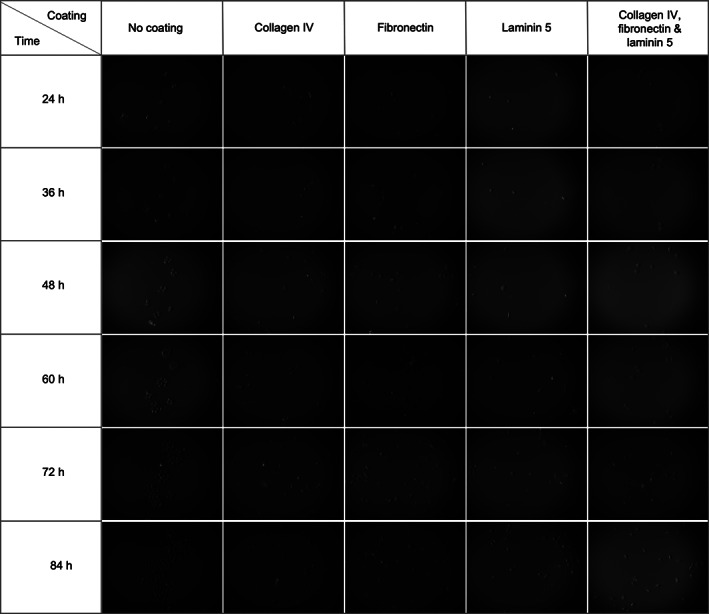
Growth, spread, and migration of keratinocytes on surfaces coated with dermal‐epidermal junction proteins: collagen IV (2 μg/cm^2^) alone, fibronectin (1 μg/cm^2^) alone, laminin 5 (2 μg/cm^2^) alone, and a combination comprising collagen IV (8 μg/cm^2^), fibronectin (4 μg/cm^2^), and laminin 5 (0.5 μg/cm^2^). An uncoated surface was used as control. Twenty‐four hours after cell seeding, the cultures were transferred to the Olympus Vivaview® microscope and live images were acquired every 30 min and are presented here at 12‐h intervals. Each experiment was performed in duplicate and representative images are shown

To further investigate the distribution and migration of keratinocytes on uncoated versus coated substrates, we performed live‐imaging analysis over a period of 84 h using the Olympus Vivaview® microscope as described in Materials and Methods. Based on the hypothesis that a complex network of DEJ molecules would promote epidermal homeostasis, we chose four representative combinations: protein coatings comprising collagen IV (2 μg/cm^2^) alone, fibronectin (1 μg/cm^2^) alone, laminin 5 (2 μg/cm^2^) alone, and a ternary combination of collagen IV (8 μg/cm^2^), fibronectin (4 μg/cm^2^), and laminin 5 (0.5 μg/cm^2^). The live images were taken every 30 min and are presented in Figure 3 as a set of representative images at 12‐h intervals. In the control condition (uncoated substrate), the cells proliferated as colonies with movement limited to their cluster space. Furthermore, the colonies formed at, generally, the same locations where the keratinocytes had originally attached. On the other hand, a very dynamic cell behavior was observed in all conditions that had been coated with the dermal‐epidermal junction proteins. In the coated wells, the keratinocytes were observed to move constantly and spread uniformly across the entire substrate instead of growing as colonies.

#### 2.1.4 Evaluation of the effect of DEJ proteins on reconstructed skin models

**FIGURE 4:**
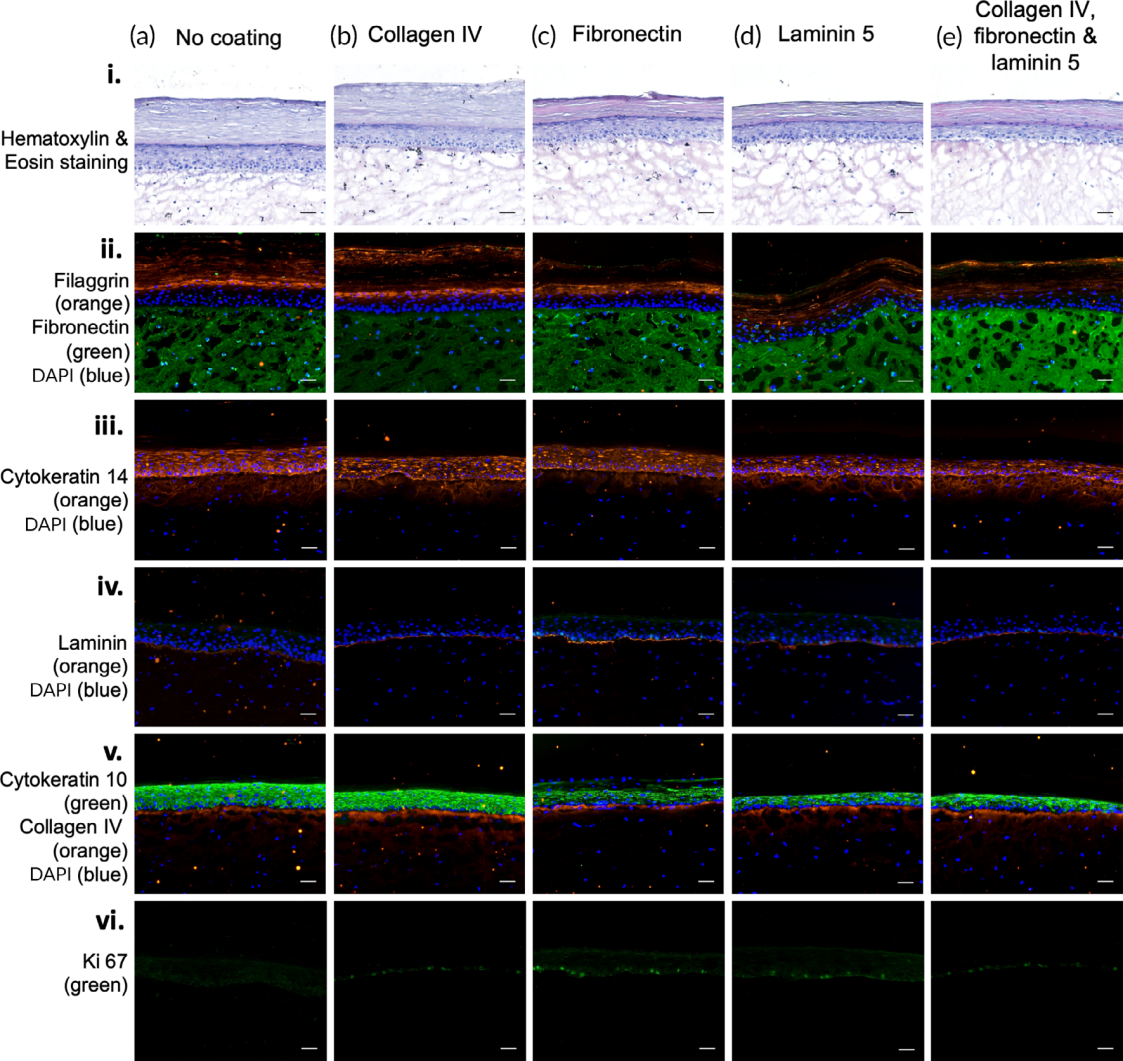
Images of reconstructed skin model including a dermal‐epidermal junction layer. Column (a): Control (only dermal and epidermal compartment); Column (b): collagen IV (2 μg/cm^2^); Column (c): fibronectin (1 μg/cm^2^); Column (d): laminin 5 (2 μg/cm^2^); Column (e): combination of collagen IV (8 μg/cm^2^), fibronectin (4 μg/cm^2^), and laminin 5 (0.5 μg/cm^2^). Row (i): Histological analysis—hematoxylin and eosin stain (10 μm sections). Rows (ii)–(vi): Analysis of skin markers by immunofluorescence (10 μm sections); Row (ii): filaggrin (orange), fibronectin (green), DAPI (blue); Row (iii): cytokeratin 14 (orange), DAPI (blue); Row (iv): Laminin 5 (orange), DAPI (blue); Row (v): cytokeratin 10 (green), collagen IV (orange), DAPI (blue); Row (vi): Ki 67 (green). The in vitro skin models were generated using a mixed population of human primary cells (fibroblasts, keratinocytes, and melanocytes) isolated from neonatal foreskin samples. Scale bar = 50 μm

The dermal layer of the reconstructed skin model was formed by human primary fibroblasts embedded in a matrix of rat tail Type I collagen. DEJ proteins were deposited on top of the dermal compartment before seeding keratinocytes to form the epidermal layer. After 14 days of exposure at the air‐liquid interface, the samples were sectioned and characterized for their morphology and expression of cell‐specific markers. In the histology analysis (H&E staining) of the control and the four conditions evaluated (Figures 4—i (a–e)), at the epidermal layer, a transition in the morphology of the cells from the basal layer to the *stratum corneum* can be observed. The keratinocytes, which present an oval shape at the *stratum basale*, progressively differentiate into enucleated flattened cells containing keratohyalin granules (a characteristic of differentiated cells), and in the final stage of the differentiation, they become corneocytes at the *stratum corneum*, where the main barrier properties of the skin are located.^29^, ^39^, ^40^, ^41^, ^42^, ^43^

The presence of skin markers throughout the epidermal layer is indicative of the expected stratification of the epidermis. The differentiated layers (*stratum granulosum* and *corneum*) are characterized by the presence of proteins associated with the cornified envelope such as filaggrin.^40^, ^44^, ^45^, ^46^, ^47^ In all conditions tested, filaggrin expression was similarly detected in the upper layers of the epidermis (Figure 4—ii (a–e)). Cytokeratin 14 and 10 are two proteins expressed, respectively, by highly proliferating undifferentiated cells^23^, ^40^, ^41^, ^45^, ^48^, ^49^, ^50^ and by cells in the suprabasal layers.^40^, ^41^, ^45^, ^50^, ^51^ As can be seen in Figure 4—iii (a–e), cytokeratin 14 was strongly detected in the basal and suprabasal layers of the epithelium, and cytokeratin 10 was detected in the suprabasal layers (Figure 4—v (a–e)). The proliferative capacity of the cells in the epidermis can be confirmed by the detection of Ki67^40^, ^52^ in the keratinocytes of the *stratum basale*. As can be seen in Figure 4—vi (a–e), only the epidermal layers generated on top of the dermal compartment coated with the DEJ proteins clearly presented proliferative keratinocytes. This could suggest that these combinations of biomaterials can support the proliferative capacity of keratinocytes for longer periods. This observation, however, remains to be further investigated. Finally, similar levels of expression of collagen IV (Figure 4—v (a–e)), laminin 5 (Figure 4—iv (a–e)), and fibronectin (Figure 4—ii (a–e)) were detected at the dermal‐epidermal junction and dermal layer in all the conditions tested.

### 2.2 Evaluation of hydrogel compositions for the reconstruction of dermal compartment of skin

#### 2.2.1 Screening of biomaterials for reconstruction of dermal compartment

**FIGURE 5:**
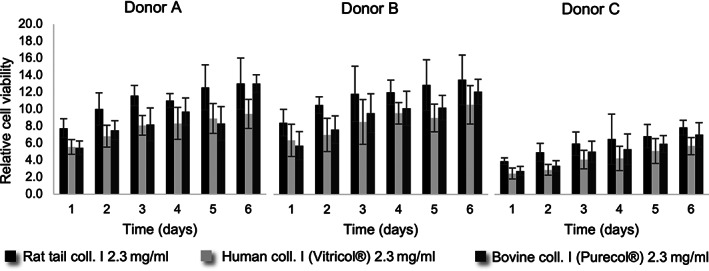
Viability of fibroblasts in hydrogels formed using Type I collagen from rat (2.3 mg/ml), human (2.3 mg/ml), or bovine (2.3 mg/ml) source. PBS was used as solvent for collagen. Cell viability was evaluated using PrestoBlueÒ solution. Cell proliferation rate was calculated by normalizing fluorescence readings of hydrogels containing cells to controls of hydrogels without cells. The results present the average ± SD of data from three independent experiments each performed in triplicate (*n* = 9)

We first evaluated the influence of the source of Type I collagen on the rate of proliferation of fibroblasts. Specifically, we tested Type I collagen from rat, human (VitroCol®), and bovine (PureCol®) sources. Similar to what was observed with the keratinocytes, there is also a difference in the rate of proliferation of fibroblasts depending on the cell source. However, as it can be inferred from Figure 5, here, the main difference seems to be related to the donor in general rather than specific age or anatomical location of the skin samples. Furthermore, these results show an influence of the source of the Type I collagen used in the hydrogels on the metabolic activity of the fibroblasts over time. Throughout the period evaluated, and among the three cell donors, the hydrogels constituting rat tail Type I collagen exhibited the highest proliferation rate, followed by the hydrogels constituting bovine and human collagens Type I, respectively.

**FIGURE 6:**
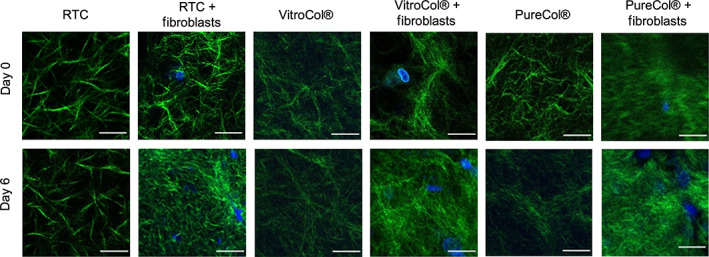
Comparison of collagen from different origins with and without fibroblasts. The top row shows images obtained on Day 0, and the bottom row shows images obtained on Day 6. The collagen fibers are shown in green, and the nuclei of the cells are shown in blue. Scale bar = 10 μm

To visualize different collagens, second harmonic generation images of the constructs with and without fibroblast were acquired at Day 0 and 6 days after seeding (Figure 6). Second harmonic generation microscopy enables label free imaging of the collagen fibers.^53^ Bovine collagen showed finer fibers of collagen compared to collagen derived from human or rat, while the rat collagen was seen to have the largest fibers. Evaluation of collagen from different origins showed no visible contraction of the fibers when fibroblasts were absent. Addition of fibroblasts to the collagen showed distinct signs of contraction on Day 6. Early signs of contraction could be detected in PureCol® samples (bovine collagen) as early as day 0 with Fibroblasts, in contrast to collagen from human and rat.

The shrinkage of the gels was measured using a Vernier caliper. While no significant differences can be found between Day 0 and Day 6 for samples without cells, the addition of fibroblasts led to a contraction of the gels to less than half their original size, from 2.0 to 0.85 and 0.94 cm (Figure S1).

**FIGURE 7:**
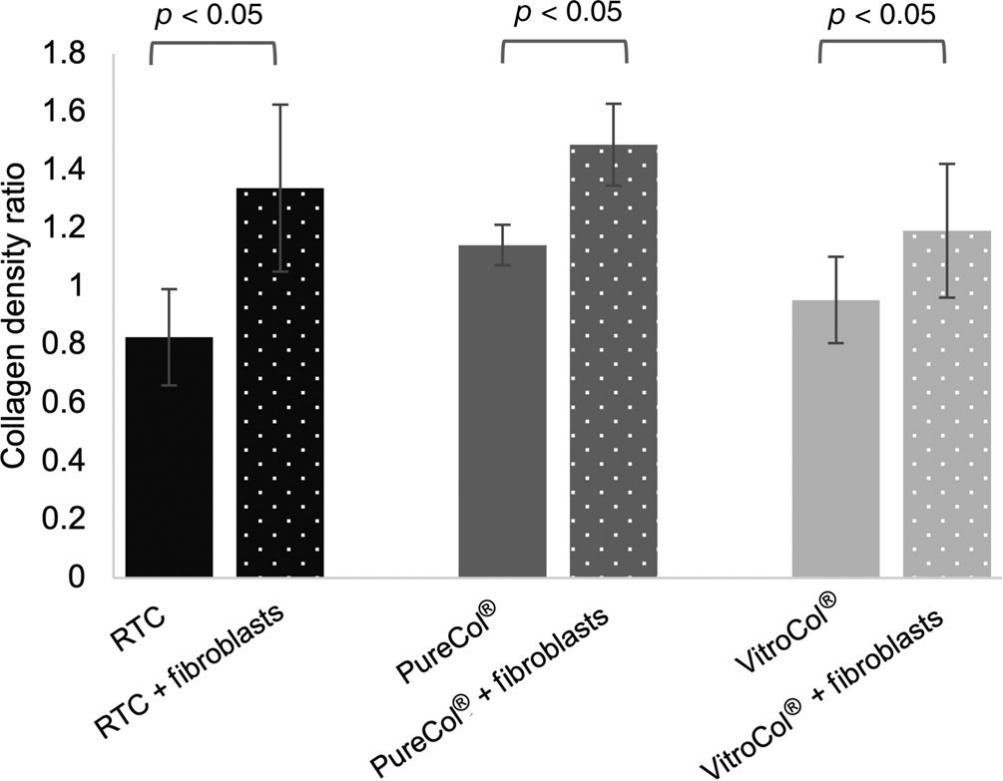
Contraction of hydrogels from different origins (Rat Tail Collagen [RTC], PureCol®, and VitroCol®) over 6 days. Each image was segmented in FIJI using Li's Minimum Cross Entropy thresholding method to calculate the area covered by collagen. To calculate the collagen density ratio, the average area percentage from Day 6 was divided by the data from Day 0. Every experiment was performed in duplicate and three images were acquired in different xyz positions for each duplicate (*n* = 6). The results present the mean ± SD of the data

To quantify the contraction of the collagen taking place on a micrometer scale, the area filled by the collagen in the images acquired using 2‐photon microscopy. The collagen area was found in each image by segmenting the images into background and collagen. Using the determined area of collagen, the average area percentage taken up by collagen was calculated. Overall, the average total area percentage taken up by the collagen network was found to be between 20% and 30% for samples on Day 0, and between 30% and 40% on Day 6 (see Figure S2). To quantify the ratio of change due to the fibroblasts, the data from Day 6 were normalized by calculating the ratio of the data from Day 6 against the data obtained from the corresponding fibroblast‐free gels from Day 0 (Figure 7). In general, the value for gels without fibroblasts was close to 1, suggesting that the density of the collagen does not change considerably. For the gels with fibroblasts, a ratio of 1.19–1.48 is seen, showing that the collagen network contracts and becomes 19%–48% denser. No significant difference was found between different collagen density ratios. The lowest collagen density ratio for hydrogels with fibroblasts was observed for VitroCol®, followed by Rat Tail Collagen and PureCol®.

**FIGURE 8:**
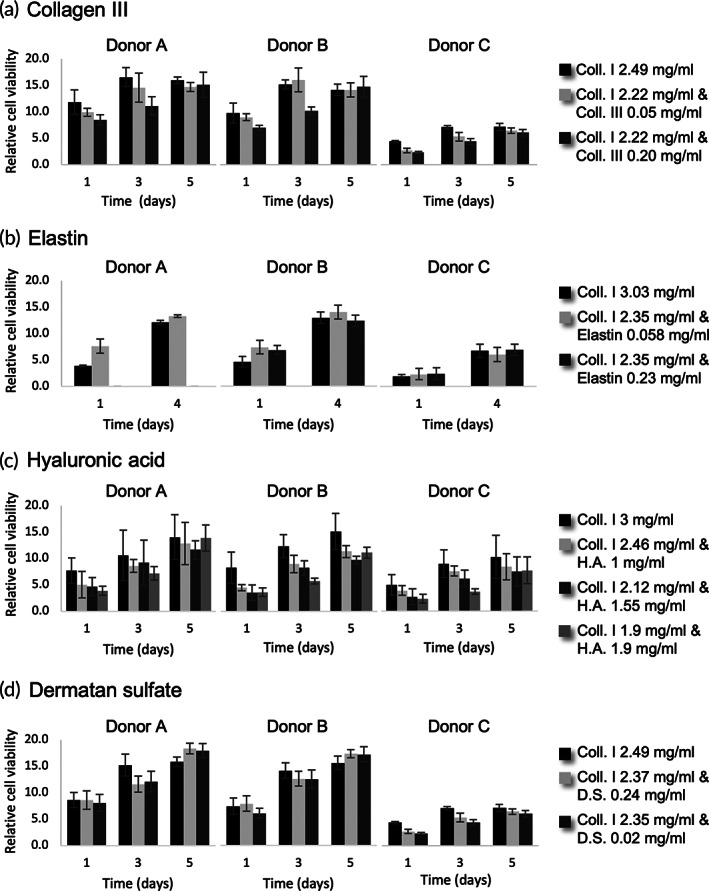
Viability of fibroblasts in hydrogels formed by collagen type I from Rat tail and (a) Type III collagen, (b) elastin, (c) hyaluronic acid, and (d) dermatan sulfate. Cell viability was evaluated using PrestoBlue® solution. Relative cell viability was calculated by normalizing fluorescence readings of hydrogels containing cells to controls of hydrogels without cells. The results present the average ± SD of the data from two independent experiments each performed in triplicate (*n* = 6)

Next, we evaluated the effect of other components (collagen Type III, hyaluronic acid, elastin, and dermatan sulfate) along with collagen Type I on the rate of proliferation of fibroblasts as shown in Figure 8.

Based on the results, inclusion of these additional molecules within rat tail Type I collagen hydrogel had no significant negative effect on the growth rate of the fibroblasts. Nonetheless, a reduction in the metabolic activity of the fibroblasts can be observed with increasing concentration of hyaluronic acid in the hydrogels (Figure 8c). Further, hydrogels containing hyaluronic acid presented visually inferior gelation compared to the control (collagen I hydrogel at equivalent concentrations). In contrast, addition of elastin to rat tail Type I collagen hydrogels visually improved the mechanical properties of the hydrogels (stiffer than control). However, due to the uniform contraction of all the hydrogels after 2 days in culture, this initial visual difference disappeared. Hydrogels composed of collagen type I and dermatan sulfate presented similar proliferation rates as the hydrogel without dermatan sulfate (Figure 8d). Nevertheless, in these 96‐well experiments a delay in the initial contraction of the hydrogel containing dermatan sulfate and an overall reduction in the final extent of contraction was observed compared to the control where the dermal scaffold was composed primarily of collagen Type I.

**FIGURE 9:**
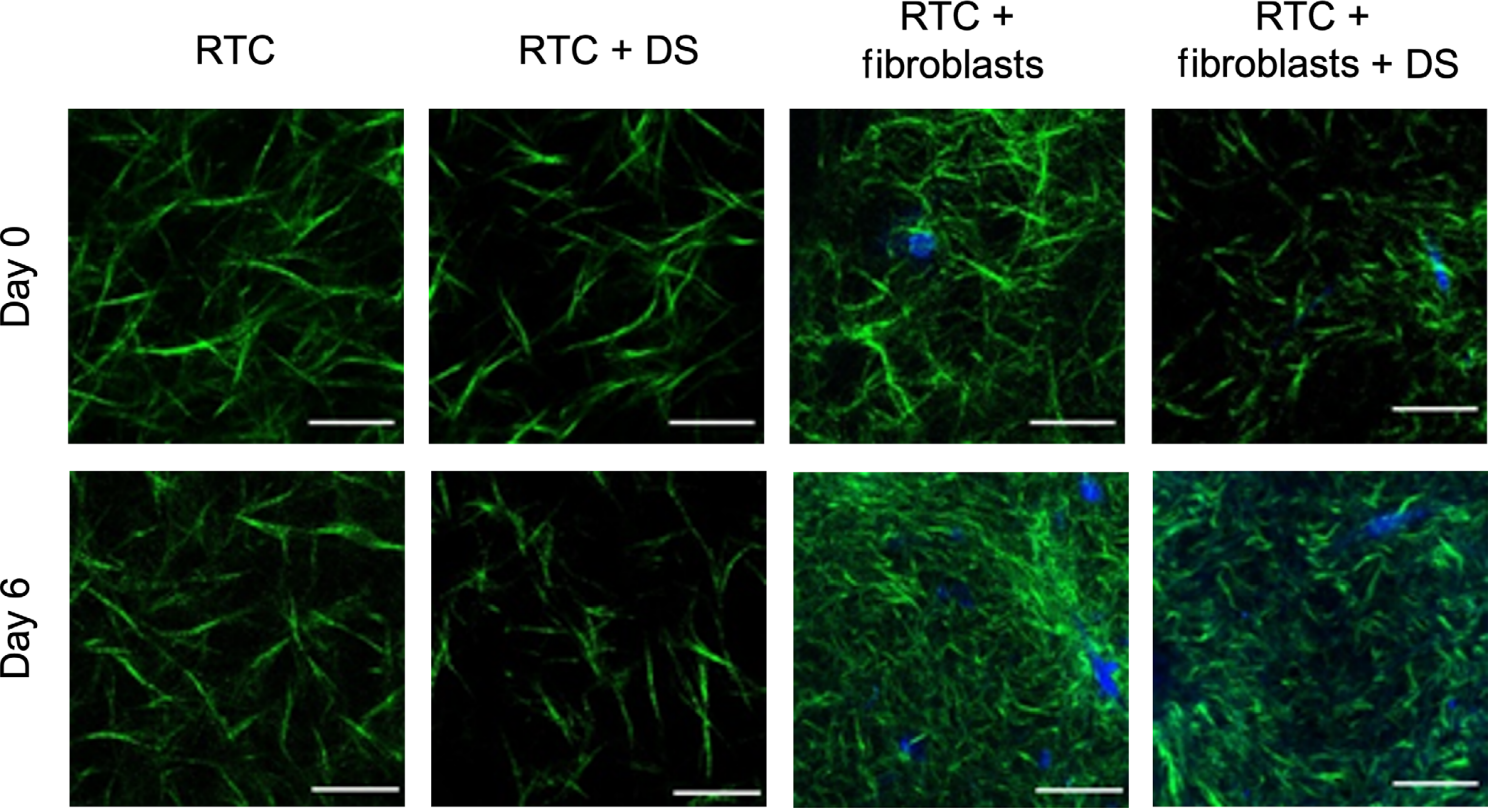
Comparison of the effect of dermatan sulfate (DS) on rat tail collagen with and without fibroblasts. The top row shows images obtained on Day 0, the bottom row shows images obtained on Day 6. The collagen fibers are shown in green and the nuclei of the cells are shown in blue. Scale bar is 10 μm

Based on these findings, we further investigated the distribution of rat tail collagen and the influence of dermatan sulfate on the contraction of the hydrogels. Imaging of the hydrogels showed sparser collagen density for samples supplemented with dermatan sulfate (Figure 9). Addition of fibroblasts showed the expected contraction of collagen fibers on Day 6 (see Figure S3); however, the addition of dermatan sulfate resulted in less densely packed hydrogels.

**FIGURE 10:**
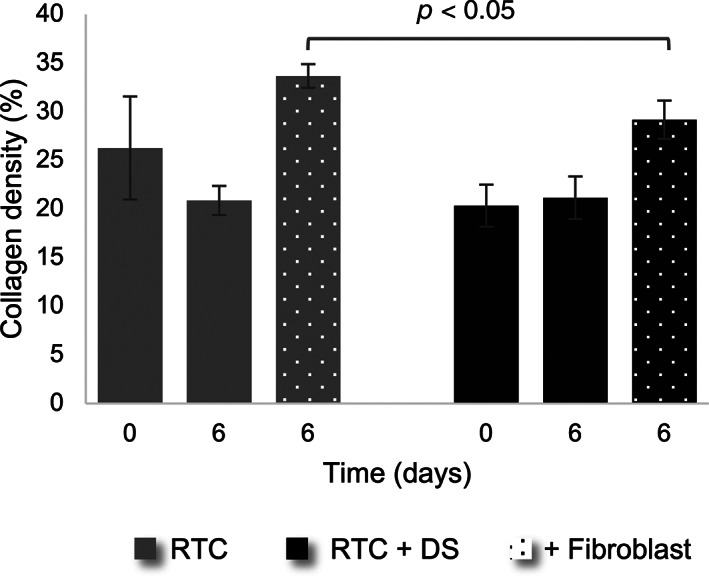
Effect of dermatan sulfate (DS) on the density of rat tail collagen (RTC) with and without fibroblasts. Each image was segmented in FIJI using Li's Minimum Cross Entropy thresholding method to calculate the area covered by collagen. Solid gray bars represent collagen density without DS; solid black bars represent the density of collagen with DS; dotted bars represent measurements of gels with fibroblasts on Day 6. Every experiment was performed in duplicate and three images were acquired in different xyz positions for each duplicate (*n* = 6). The results present the mean ± SD of the data

To quantify the density of the collagen network, the area filled by collagen in the images was calculated. An overall lower collagen density for samples including dermatan sulfate was observed (Figure 10). A significant difference in the mean was found between gels packed with fibroblasts and with or without dermatan sulfate. The effect on the microscopic scale can be seen from Day 0. A measurement of the diameter of the hydrogels showed no significant difference in contraction.

#### 2.2.2 Evaluation of the effect of dermal hydrogel composition on reconstructed skin models

**FIGURE 11:**
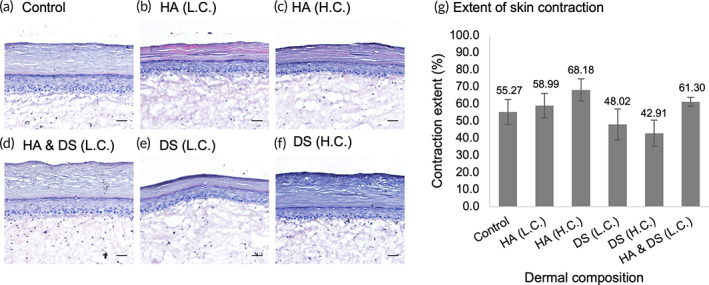
Histology analysis of reconstructed skin models generated with dermal composition containing hyaluronic acid (HA) and dermatan sulfate (DS) at low and high concentrations (L.C. and H.C.). (a) Control: Rat tail collagen type I (2.52 mg/ml); (b) HA (L.C.): Rat tail collagen Type I (2.51 mg/ml) and hyaluronic acid (0.025 mg/ml); (c) HA (H.C.): Rat tail collagen Type I (2.4 mg/ml) and hyaluronic acid (0.24 mg/mL); (d) HA (L.C.) & DS (L.C.): Rat tail collagen Type I (2.4 mg/ml), hyaluronic acid (0.025 mg/ml) and dermatan sulfate (0.024 mg/ml); (e) DS (L.C.): Rat tail collagen Type I (2.51 mg/ml) and dermatan sulfate (0.025 mg/ml); (f) DS (H.C.): Rat tail collagen Type I (2.4 mg/ml) and dermatan sulfate (0.24 mg/ml). The in vitro skin models were generated using a mixed population of human primary cells (fibroblasts, keratinocytes, and melanocytes) (hematoxylin and eosin stain—10 μm cryosections). Scale bar = 100 μm. (g) Percent skin contraction (xy direction), calculated as the ratio of contracted area (initial–final surface area) to initial surface area of the skin samples (112 mm^2^). The results represent the average ± SD of the data from three independent experiments each performed in triplicate (*n* = 6) for samples: Control, HA (L.C. & H.C.), DS (L.C. & H.C.), and two independent experiments each performed in duplicate (*n* = 4) for samples: HA and DS (L.C.)

Histological analysis of the reconstructed skin samples generated with different dermal hydrogels (Figure 11) shows that all dermal compositions tested were able to support epidermal proliferation and differentiation. Proliferative keratinocytes from the basal layer were observed to progressively differentiate to form the *stratum corneum*. Furthermore, when evaluating the effect of the hydrogel composition on the extent of tissue contraction (xy direction) it can be seen that hyaluronic acid, at both concentrations tested, was unable to reduce skin contraction compared to the control in (Figure 11g). Dermatan sulfate, on the other hand, reduced contraction up to 28% ([final surface area of control−final surface area of sample]/final surface area of control) in a concentration‐dependent manner.

## 3 DISCUSSION

Reconstructed skin models have been developed and studied for more than two decades. These models have been extensively characterized for their morphology, functionality, and similarity to the human skin.^12^, ^23^, ^28^, ^29^, ^40^, ^42^, ^47^, ^51^ They have been validated as alternative methods to animal models for the safety assessment of substances and explored as relevant tools for efficacy evaluation of topically applied products.^54^, ^55^, ^56^, ^57^ Furthermore, the field of regenerative medicine has evolved exponentially toward the development of skin grafts that could be used for clinical applications.^27^, ^58^, ^59^ Nonetheless, engineered skin models still fail to recapitulate the complexity of the native tissue in terms of cellular diversity (e.g., lacking melanocytes, neural and immune cells), presence of adnexal structures (e.g., lacking hair follicle, sebaceous, and sweat glands), and vascularization. Furthermore, the currently available reconstructed skin models employ a very limited array of matrix proteins and scaffold biomaterials compared to the diversity of biomolecules present in the human skin, which are important for tissue homeostasis.^40^, ^51^, ^54^, ^60^, ^61^

In this study, we investigated the effect of employing a more complex and defined matrix on the growth and support of cells in 2D as well as on promoting proper development of a full thickness 3D skin model. Using this approach, we first analyzed the impact of collagen IV, fibronectin, and laminin 5, found at the dermal‐epidermal function of the native skin, on the growth of keratinocytes in a monolayer. We observed that some of these proteins or their combinations promoted the proliferation of keratinocytes while others inhibited their growth. More specifically, among the conditions tested, collagen IV at 2 μg/cm^2^ induced the highest rate of proliferation of the keratinocytes isolated from skin tissues obtained from three independent donors. In contrast, fibronectin at 1 μg/cm^2^ inhibited the proliferation of keratinocytes. These findings are in agreement with those reported by Dawson et al., which demonstrated that collagen IV surface coating promoted proliferation of keratinocytes while keratinocytes grown on plastic surface coated with fibronectin proliferated more slowly than those seeded directly on plastic surface.^21^ One possible explanation is that in keratinocytes, fibronectin receptors are trypsin‐sensitive, which suggested that, in vitro, their adherence to fibronectin depends on de novo synthesis of this receptor.^25^ The need for the synthesis of new receptors prior to the adherence of the cells to the surface coated with fibronectin could explain the decreased proliferation rate. A few combinations showed variability in proliferation response, which may be attributed to factors of genotypic and/or epigenetic origin that while interesting were beyond the scope of this study and will be probed in future studies.

It was additionally observed (data not shown) that in the control wells (uncoated substrates), keratinocytes preferred to proliferate as isolated colonies while on the coated substrates the cells spread more uniformly as a monolayer. Lamb and colleagues have previously observed that keratinocytes grown in media supplemented with serum and high concentration of calcium were highly motile compared to serum‐free and low calcium condition, demonstrating the influence of growth conditions on cell behavior.^49^ Similarly, Huang and colleagues showed that media composition not only affects cell morphology and proliferation but also influences colony formation.^62^ To further investigate the colony formation behavior, we performed a live analysis of the movement and distribution of keratinocytes over a period of time. We demonstrated that keratinocytes cultured on uncoated substrates grow as colonies while cells grown on substrates coated with the proteins from the dermal‐epidermal junctions present a dynamic behavior and uniform spreading. This confirmed our observation that, beyond regulating cell proliferation, these proteins promote cell migration and result in uniform cell distribution. In vivo, progression of keratinocytes through the cell cycles is affected by the degree of differentiation, matrix adhesion, and growth factors.^63^ The proper balance between the proliferation and differentiation of keratinocytes, which presents a turnover of around 30 days, results in tissue homeostasis.^64^, ^65^ In vitro, the loss of a physiological state and inclusion of artificial growth conditions favors a proliferative profile that contributes to cellular expansion in monolayer.^63^, ^64^, ^66^ Furthermore, media composition, such as presence of serum and antibiotics, concentration of calcium and growth factors, or culture conditions, can modulate cell behavior in monolayer as well as influence outcomes in 3D in vitro models.^49^, ^62^, ^67^ We hypothesized that the inclusion of a DEJ layer in the skin reconstruction protocol could accelerate early keratinocyte proliferation and distribution contributing to the formation of a uniform confluent monolayer prior to the beginning of the cell differentiation process when exposed to the air‐liquid interface. Furthermore, we believe that this characteristic could contribute toward accelerating the host integration of skin graft placed in wounds as it is known that proliferation and migration of keratinocytes are fundamental for tissue reepithelization.^58^, ^66^ Considering this hypothesis and understanding that beyond cell attachment and cell proliferation rate, these DEJ proteins must promote proper keratinocyte differentiation, we evaluated the effect of four distinct protein compositions in the skin reconstruction context. Following 14 days of tissue maturation, we did not observe any significant morphological differences between the control and test conditions. Nevertheless, the staining with Ki67 suggested that these compositions could indeed be supporting prolonged keratinocyte proliferation, which remains to be further investigated. Based on these results, we speculate that the in vitro period required for the maturation of the tissue could be long enough that any early positive or negative effect of the inclusion of these proteins at the dermal‐epidermal junction would be compensated over time by the innate capacity of cells to produce extracellular matrix molecules and reach homeostasis. Similarly, Boehnke and colleagues observed that the reconstructed skin models generated in their experiments, presented a convergent development process, which culminated in tissues with similar characteristics, independently of the initial parameters tested (cell number, pre‐cultivation protocol, media composition).^29^ Nonetheless, we posit that an early enhancement in proliferation and migration of epidermal cells, promoted by the presence of a fabricated dermal‐epidermal junction, could be beneficial for the reduction of maturation time of in vitro models or even in cases where a quick recovery of the epidermal layer is desired, such as wound engraftment for regenerative medicine applications.

To investigate the effect of the matrix composition on embedded fibroblasts, we evaluated the rate of proliferation of the dermal fibroblasts through the analysis of the metabolic activity of the cells within the hydrogels maintained in submerged culture. Similar to what was observed with keratinocytes, fibroblasts exhibit a rate of proliferation that is source dependent, independent of the hydrogel composition. However, while keratinocytes isolated from neonatal foreskin had a higher rate of proliferation compared to cells isolated from adult skin, the differences in the proliferation of fibroblasts could not be associated with age, gender or anatomical location, but it was rather donor specific. Accordingly, Lange et al. showed that optimal culture conditions to generate skin models may differ depending on the cell source.^68^ The donor‐to‐donor variability is a key point in the development of tissue engineered models using primary cells for regenerative medicine and fabrication of in vitro models. These observations demonstrate the importance of using a representative cell population for the optimization of biomaterials for tissue engineering.

Studying the influence of the source of the Type I collagen used to generate the scaffold, we observed the highest metabolic activity from fibroblasts embedded in rat tail Type I collagen hydrogel, followed by bovine and human sourced collagen. Considering the principle of the methods used (cell viability based on the metabolic activity of cells), this could indicate that indeed the overall number of metabolically active cells is different, or that the cells within different hydrogels present distinct metabolic activity. The latter could be associated with the nature of the scaffold per se or the presence of other components, such as growth factors and other proteins, carried over during the extraction process employed for producing the various collagens.

Comparison of collagen from three different origins showed contraction of gels when fibroblasts were included in the gel irrespective of the source of the collagen. On the macroscopic scale, the change in contraction was not significantly different between the three sources. On the micrometer scale, the second harmonic generation images of collagen showed first signs of contraction within a few hours after seeding, thus agreeing with previously published macroscopic measurements of collagen contraction.^69^, ^70^ Quantification of the images of collagen showed similar overall collagen densities; however, there were differences in the collagen fiber size, with bovine and human collagen having finer fibers than rat tail collagen. The collagen density ratios showed differences between the three gels after 6 days of culture with fibroblasts. The largest change was detected in gels comprising bovine (PureCol®) collagen while a significant but smaller change was measured in the human and rat tail collagen. It is interesting to note that the viability test of the cells in the collagens showed similar behaviors for the human and rat collagens, the viability of the cells in the bovine collagen was considerably lower. Further work is needed to determine if this is due to the collagen fiber size and/or density, or other factors.

Analysis of the effect of dermatan sulfate showed no significant difference in the diameter between samples with or without dermatan sulfate. However, on the micrometer scale, a lower collagen density can be observed in all samples including dermatan sulfate. This may pinpoint to the influence of dermatan sulfate on the gels, which could already come into effect from the moment of polymerization. Furthermore, the addition of dermatan sulfate may have adverse effects on the production of collagen by fibroblasts. With the methods used in this study, no clear distinction can be made between collagen produced by fibroblasts, and collagen from the hydrogels, thus not allowing a separation between the increase in collagen production from the fibroblasts and the increase due to fibroblast contraction. Imaging of the fibers, with dermatan sulfate, shows alterations of the network density as early as Day 0. Previous studies have reported alterations of the fibril density and width from the addition of dermatan sulfate.^71^ Modification of the collagen organization has previously also been shown by Stuart and Panitch, showing that addition of dermatan sulfate led to an increased amount of void space.^72^ The study was, however, performed on cell‐free gels. Taken together with the results presented in this study, we suggest that the effect of dermatan sulfate may not be cumulative, meaning the relative amount of void space present in the gel is determined from the moment of polymerization, and the relative contraction of the gel is only minimally influenced by the addition of dermatan sulfate. The results do however suggest a more regulated organization and polymerization of the fibers, showing more coherent results in gels with dermatan sulfate compared to gels without it.

Regarding the more complex scaffold generated by the mixture of Type I collagen and other matrix molecules, when we compared each condition that contains hyaluronic acid to its respective control in terms of collagen Type I dilution, we can infer that the decrease on the metabolic activity of fibroblasts observed can be associated with the lower concentration of type I collagen and not the presence of the hyaluronic acid. Previously, and consistent with our findings, Kreger and Voytik‐Harbin also observed that addition of hyaluronic acid to collagen I hydrogels did not significantly change the proliferation of human dermal fibroblasts embedded in the gels.^73^

Independent of the hydrogel compositions, we observed only a small increase in the metabolic activity or total number of cells over the period evaluated. In the native tissue and under steady state conditions, the mitotic activity of adult fibroblasts in most locations is very low compared to fibroblasts grown in vitro or even epidermal keratinocytes in the *stratum basale*.^74^ Considering that our goal is to generate a skin model that mimics healthy human tissues, the lack of significant increase in the rate of proliferation of the fibroblasts within the 3D hydrogel is normal and expected. We concluded that the formulated hydrogels tested were non‐cytotoxic to the fibroblasts and did not induce abnormal cell growth.

One of the main challenges in the fabrication of the reconstructed skin models in vitro is the contraction (x, y, and z directions) that most of the constructs developed undergo during the submerged condition and more prominently when exposed to the air liquid interface.^29^, ^75^, ^76^ It has been shown that the contraction of skin models in vitro as well as hydrogels without epidermal cells is influenced not only by the fibroblast density and culture period but also by the concentration of collagen type I.^77^ This is one of the aspects that limits the life span of most in vitro models to a maximum of 8 weeks in culture.^35^ Over the years, several groups have been studying the mechanisms underlying this phenomenon and investigating strategies to modulate and control, but with limited success. Some of these successful strategies have used stabilized scaffolds to support the development of well‐differentiated and mechanically stable skin models that could be maintained for up to 12 weeks at the air liquid interface.^28^, ^29^ Boehnke and colleagues demonstrated that a hyaluronic acid esterified with benzylic alcohol scaffold provided counteracting forces to prevent skin contraction but also supported the synthesis of de novo extracellular matrix.^29^ Similarly, in the work done by Mewes and colleagues, fibroblasts embedded on the stabilized collagen scaffold secreted extracellular matrix proteins and created a network of elastic‐fibers similar to that observed in the human skin, which supported tissues homeostasis for 51 days.^28^ We speculated that the inclusion of the glycosaminoglycans such as hyaluronic acid and dermatan sulfate could reduce the degree of tissue contraction by controlling the water‐binding capacity or ECM modification^9^, ^78^, ^79^, ^80^, ^81^ In our experiments, the poor gelation of the gels containing hyaluronic acid could be a result of the absence of crosslinking of the hyaluronic acid. Furthermore, the hydrogels containing hyaluronic acid were not able to reduce the contraction of the tissue samples. Accordingly, Kreger and Voytik‐Harbin also demonstrated that the inclusion of hyaluronic acid in collagen Type I hydrogels did not affect skin contraction by fibroblasts.^73^ However, the hydrogels containing dermatan sulfate and Type I collagen reduced the contraction of the samples compared to the control, which could be associated with higher water retention (substrate‐bound and free water).^80^ Also, the combination of the in vitro condition and the presence of dermatan sulfate could be inducing fibroblasts differentiation into myofibroblasts. This phenotype displays exacerbated ECM production and presents a contractile apparatus that helps them to remodulate the ECM, which is fundamental for physiological tissue repair.^81^, ^82^ In vitro, the control of ECM composition and structural modification by differentiated fibroblasts could also influence tissue integrity and contraction profile. Considering both hypothesis, the inclusion of dermatan sulfate could provide the right condition to promote de novo extracellular matrix production and improve the mechanical properties.^32^Based on these results, we infer that dermatan sulfate can modulate skin contraction while promoting proper tissue homeostasis.

Finally, analyzing the skin contraction results from several independent experiments (data not shown) we also observed considerable variability in the extent of skin contraction in the constructs with the same dermal composition. This could be associated with the specific combination of cells used in each of the independent experiments. Reconstructed skin models were generated using human primary cells isolated from skin samples discarded during surgery, and consequently the number of cells obtained from these samples is finite and their life span in culture is also reduced compared to immortalized cell lines. This implies that there is a limited supply of each cell type and, as consequence, it is frequently necessary to use different combinations of cells in the experiments. For example, it has been shown that keratinocytes isolated from African and Caucasian skin exhibit significant differences in their stratification and differentiation when included in reconstructed skin models.^83^ The differences observed here highlight, once more, the importance of using a representative population of cells in tissue reconstruction. This would not only substantiate the study in terms of observations that can be made based on specific cell populations but can also reduce the variability in these observations.

The data collected in this work could support the optimization of skin reconstruction protocols by better modulating the tissue maturation process as well as rheological properties of gel‐based models.

## 4 MATERIALS AND METHODS

### 4.1 Isolation and culture of primary cells from human skin

Normal human skin cells were isolated from donated skin samples (1 adult female breast skin sample and 12 neonatal foreskin samples) obtained from Dr. George Xu at University of Pennsylvania and Dr. David M. Owens at Columbia University under protocols approved by the respective institute's Review Board. Cells were isolated as described previously.^55^, ^84^, ^85^ Normal human epidermal keratinocytes (NHEK) were cultured in KGM® Gold Bullet Kit medium (Lonza, #192060) supplemented with isoproterenol hydrochloride 10^−6^ M (Sigma‐Aldrich, #I6504). Fibroblasts were cultured in Dulbecco's Modified Eagle Medium (DMEM) (Gibco™ by Life Technologies, #11995) supplemented with 10% fetal bovine serum (FBS) (Atlanta Biologicals, #S115500H) and melanocytes were maintained in Medium 254 (Gibco™ by Life Technologies, #M254500), supplemented with Human Melanocyte Growth Supplement (HMGS) (Gibco™ by Life Technologies, #S0025). Fibroblasts and melanocytes were maintained in a humidified chamber at 37°C containing 5% CO_2_, and keratinocytes were maintained at 37°C and 7.5% CO_2_. Cells were sub‐cultured by treatment with 0.05% trypsin–EDTA (Gibco™ by Life Technologies, #25300‐054) when they reached approximately 80% confluency.

### 4.2 Reconstruction of the skin dermal‐epidermal junction

To mimic the dermal‐epidermal junction (DEJ), the basement membrane proteins collagen IV (C) (human placenta) (Sigma‐Aldrich, #7521), fibronectin (F) (human placenta) (Sigma‐Aldrich, #F0895), and laminin 5 (L) (human placenta) (Sigma‐Aldrich, #L7264), at 2 different concentrations, were screened alone and in combination using the rate of proliferation of keratinocytes as a quantitative endpoint. The protein concentrations used for coating the seeding surface were C: 2 μg/cm^2^ (C1) and 8 μg/cm^2^ (C2), F: 1 μg/cm^2^ (F1) and 4 μg/cm^2^ (F2), and L: 0.5 μg/cm^2^ (L1) and 2 μg/cm^2^ (L2). The coating solutions were added to a 96‐well plate and incubated overnight at 4°C. On the next day, after 1 h of incubation in humidified chamber at 37°C, excess solution (30 μl) was removed from the wells and the keratinocytes were seeded at a density of 700 cells/well. The cells were maintained in a humidified chamber at 37°C and 7.5% CO_2_ for 4 days with daily media change. At Day 4, we performed a live/dead assay to quantify cell growth. Calcein‐AM (green dye = live cells) (Invitrogen™, #C3099) and propidium iodide (red dye = dead cells) (Invitrogen™, #P3566) were added to the wells and incubated for 40 min. The ArrayScaN™ XTI was used to image the wells and the image analysis provided the total number of live and dead cells for each test condition.

#### 4.2.1 Time‐lapse videomicroscopy of keratinocytes seeded on surface coated with proteins from the dermal‐epidermal junction

Keratinocytes were seeded on 35 mm optically enhanced culture dish (Ibidi®, 81,156) coated with dermal‐epidermal junction bioinks selected from the high‐throughput screening experiments. The compositions chosen were collagen IV (2 μg/cm^2^), fibronectin (1 μg/cm^2^), laminin 5 (2 μg/cm^2^), and a combination of collagen IV (8 μg/cm^2^), fibronectin (4 μg/cm^2^), and laminin 5 (0.5 μg/cm^2^). A non‐coated dish with PBS was used as control. The dishes were incubated overnight at 4°C. On the next day, the plates were transferred to a humidified chamber at 37°C for 1 h prior to seeding the keratinocytes (18,000 cells/dish). The cells were maintained in humidified chamber at 37°C and 7.5% CO_2_ for 24 h. To analyze the distribution and movement pattern of the keratinocytes seeded on top of the different coating compositions, the dishes were imaged with an Olympus VivaView® (10×) for 84 h at 2 frames/h.

### 4.3 Reconstruction of the skin dermal layer

Hydrogels composed of collagen Type I [rat tail (Corning®, #354236), human (VitroCol®—Advanced Biomatrix, #5007) and bovine (PureCol®‐ Advanced Biomatrix, #5074)], collagen Type III (human collagen Type III) (Advanced Biomatrix, #5021), hyaluronic acid sodium salt (recombinant) (Sigma‐Aldrich, #53747), elastin (human recombinant tropoelastin) (Advanced Biomatrix, #5052), and dermatan sulfate (porcine intestinal mucosa) (Sigma‐Aldrich, #C3788) were evaluated for supporting the viability of fibroblasts. Hydrogels containing (1.5 × 10^5^ cells/ml) or devoid of fibroblasts (control) were added (100 μl) to individual wells of a 96‐well plate. The gels were maintained in humidified chamber at 37°C and 5% CO_2_ for up to 7 days with daily media change. On each day, one set of plates was used to perform a metabolic assay. The gels were carefully transferred to a new plate, and a solution of PrestoBlue® reagent (Invitrogen, #A13261) in DMEM supplemented with 10% FBS (1:9) was added to the wells. Following incubation for 5 h in PrestoBlue®, the supernatant was transferred to a new plate and the fluorescence was measured using a plate‐reader (560/590 nm). The metabolic activity was calculated by normalizing fluorescence readings of hydrogels containing cells to controls of hydrogels without cells.

#### 4.3.1 Characterization of Type I collagen fibers

For the characterization of collagen fibers, dermal hydrogels were prepared by mixing 750 μl collagen with 100 μl of 10X TR Buffer (0.05 M NAOH (Sigma, #S2770), 0.26 M NaHCO_3_ (Sigma, S8761), 200 mM HEPES (Sigma, #H3375), 100 μl 10X Ham‐F12 (Gibco, #21700‐026), and 50 μl of cell suspension in FBS for a final cell concentration of 15 × 10^4^ cells/ml and the final collagen concentration was adjusted to 2.3 mg/ml using PBS. For hydrogels without cells, 50 μl was added instead of the cell suspension. For experiments involving Dermatan sulfate (Sigma, #C3788), the sodium salt was diluted at 5 mg/ml in MilliQ and the dilution was added to the hydrogel mixture for a final mass of dermatan sulfate equalling 10% of the final mass of collagen used. One milliliter of the hydrogel was added per μ‐Dish (Ibidi, #81158) and the plates were incubated for 30 min at 37°C and 5% CO_2_. After gelation, 1.5 ml of culturing media was added on top of the gels and changed every other day. For image acquisition, the nuclei were stained using Hoechst 33342 (Invitrogen, H3570) for 10 min. The media was replaced using culturing media prepared with phenol‐free DMEM (Sigma, D1145). The images were acquired using a custom‐build 2‐photon microscope based on the Nikon Eclipse Ti‐E Inverted microscope system described here.^86^ The excitation laser was Spectra‐Physics Mai‐Tai DeepSee Tuneable Ultrafast Laser tuned to 800 nm. The microscope was controlled using Micromanager, and VistaVision (ISS). The emission signal was split by a beamsplitter (460 LP filter), the DAPI signal was detected using a (494/20 nm bandpass filter) while the SHG from the collagen was detected using a 405/14 nm bandpass filter. The area covered in collagen was calculated in FIJI^87^ by segmentation using Li's Minimum Cross Entropy thresholding method and subsequent measurement of the average area percentage.

### 4.4 Reconstruction of human skin tissue

To generate the reconstructed skin models, we started by depositing the dermal solution (800 μl) with specific hydrogel compositions and 1.5 × 10^5^ fibroblasts/ml on polyester inserts (12 mm ø, 0.4 μm pore size) (Corning® Transwell®, #3460). On top of the polymerized dermal compartment, we deposited the epidermal layer, which is formed by keratinocytes (2.5 × 10^5^ cells/sample) and melanocytes (0.25 × 10^5^ cells/sample) in RAFT: KGM Gold BulletKit (1:1) media. Alternatively, following dermal polymerization and 1 h before seeding the epidermal cells, we deposited a thin coating of selected DEJ proteins. The RAFT medium was prepared by adding DMEM and Hams‐F12 medium (Gibco™ by Life Technologies #21700–075) (3:1) with 10% FBS and supplements (10 ng/mL human recombinant epidermal growth factor (EGF) (Sigma‐Aldrich, #E9644), 5 μg/ml insulin (Sigma‐Aldrich, #I2643), 0.4 μg/ml hydrocortisone (Sigma‐Aldrich, #H4881), 0.1 nM cholera toxin (Sigma‐Aldrich, #C8052), and 5 μg/ml transferrin (Sigma‐Aldrich, #T1147). After 24 h under submerged conditions, the constructs were raised and maintained at the air‐liquid interface for 14 days to allow the complete stratification and differentiation of the keratinocytes from the *stratum basale*.

#### 4.4.1 Analysis of surface area of reconstructed skin samples

Following 14 days at air‐liquid interface, the samples were photographed, and the surface area of the samples was measured using ImageJ software (ImageJ, U.S. National Institutes of Health, Bethesda, Maryland, USA) and numerically compared. The contraction extent (xy direction) was calculated as the ratio of contracted area (initial minus final surface area) and initial surface area of the skin samples (112 mm^2^) multiplied by 100 (%).

#### 4.4.2 Histological and Immunohistochemical characterization of reconstructed skin model

The reconstructed skin samples were embedded in Tissue‐Tek O.C.T. (Sakura® Finitek, #4583), cryopreserved and then sectioned (10 μm) using a HM505E cryostat. For histological analysis with hematoxylin & eosin staining, the frozen sections were maintained at room temperature for at least 4 h prior to hydration in water (30 min). The hydrated sections were stained with hematoxylin for 2 min, washed with tap water for 5 min and then stained with Y eosin for 9 min.^88^ The sectioned tissues were sequentially immersed in ethanol solution (50%, 70%, 90%, 95%, 100% I, 100% II, 100% III) each for 5 min. After dehydration in ethanol, the slides were immersed in xylol twice for 5 min each and then sealed with coverslip using DPX mount medium (Sigma, #06522). To perform the immunofluorescence analysis, the slides containing the frozen sections of the skin samples were maintained at room temperature for at least 4 h prior to fixation with cold acetone (−20°C) for 10 min. The tissue was then rehydrated with PBS for 15 min and subsequently immersed for 10 min in a solution of PBS and goat serum (10%) to saturate the fixation sites. The samples were permeabilized by briefly immersing the slides in a 0.05% Tween 20–solution in PBS. The immunolabeling assay was carried out using the following antibodies: anti‐cytokeratin 10 (Abcam Cambridge, UK—1421, dilution 1:200), anti‐cytokeratin 14 (Abcam Cambridge, UK—7800, dilution 1:200), anti‐collagen IV (Abcam Cambridge, UK—6311, dilution 1:200), anti‐filaggrin (Abcam Cambridge, UK—24584, dilution 1:100), anti‐Ki67 (Bd—6100968, dilution 1:100), anti‐collagen IV (Abcam Cambridge, UK—6311, dilution 1:200), anti‐fibronectin (Abcam Cambridge, UK—32419, dilution 1:150), and anti‐laminin 5 (Abcam Cambridge, UK—78286, dilution 1:100). The following secondary antibodies with fluorophores were used for detection: anti‐mouse Alexafluor® 488 (goat) anti‐mouse (Invitrogen a11001, dilution 1:200), anti‐rabbit Alexafluor® 488 (goat) anti‐rabbit (Invitrogen a11034, dilution 1:200), and Alexafluor® 555 (donkey) anti‐mouse (Invitrogen a31570, dilution 1:200). The slides were mounted using Vectashlied® with DAPI (nuclear blue stain). All images were obtained and analyzed using an Olympus IX51 Fluorescence Microscope.

## 5 CONCLUSION

Despite being considered equivalent to human skin, reconstructed skin models still fail to reproduce the same level of complexity and physiological characteristics of the native tissue. These gaps limit not only their application as platforms for toxicological screening of substances in vitro but also their use as grafts for the treatment of wounds due to impaired host integration. In the present study, we demonstrate that proteins from the DEJ (collagen IV, laminin 5, and fibronectin) can modulate not only the proliferation of keratinocytes grown as a monolayer but also their migration and distribution in 2D. More specifically, we show that collagen IV can singularly induce a higher proliferation rate compared to the other proteins, individually or in combination. These results suggest that the proper combination of dermal‐epidermal junction proteins could support skin graft integration as well as modulate cellular turnover in in vitro models. However, in the 3D context of the fabricated skin, we observed that the cells can produce these proteins and form a DEJ independent of the deposition of any of the proteins initially, suggesting that a positive effect could be limited to early stages of tissue maturation In the investigation of the effect of components from the dermal layer (collagen I and III, elastin, hyaluronic acid, and dermatan sulfate), the primary influence on the viability of fibroblast was attributed to the source of the collagen type I (rat tail, human and bovine) used as a scaffold material. Furthermore, the incorporation of dermatan sulfate in the dermal matrices lead to a reduction of the contraction of skin samples compared to the control samples whose dermal scaffold is composed primary of collagen type I. Finally, we observed differences in response of cells from different donors (proliferation and metabolic activity).

Our results highlight the relevance of the biomaterial composition and cell source to the development of complex reconstructed skin models. Biomaterial enrichment, as well as culture optimization, could be a key step for supporting cellular diversity in tissue models and hence, truly achieving tissue complexity. Furthermore, we believe that these findings along with work done by other colleagues in the field of tissue engineering can contribute, for example, to the design of bioinks to be used to 3D bioprint a new generation of physiologically more relevant reconstructed skin models.

## AUTHOR CONTRIBUTIONS

**Katharina Kaiser:** Data curation (supporting); formal analysis (supporting); investigation (supporting); methodology (supporting); writing – original draft (supporting). **Tânia Baltazar:** Conceptualization (supporting); methodology (supporting). **Luiza Motter Catarino:** Data curation (supporting); formal analysis (supporting); investigation (supporting); methodology (supporting). **Jonathan Brewer:** Conceptualization (supporting); formal analysis (supporting); project administration (supporting); resources (supporting); writing – original draft (supporting). **Carolina Motter Catarino**: Conceptualization‐Lead, Data curation‐Lead, Formal analysis‐Lead, Funding acquisition‐Supporting, Investigation‐Lead, Methodology‐Lead, Project administration‐Lead, Writing – original draft‐Lead. **Pankaj Karande:** Conceptualization‐Supporting, Data curation‐Supporting, Formal analysis‐Supporting, Funding acquisition‐Lead, Investigation‐Supporting, Methodology‐Supporting, Project administration‐Supporting, Resources‐Lead, Writing – original draft‐Supporting.

### PEER REVIEW

The peer review history for this article is available at https://publons.com/publon/10.1002/btm2.10297.

## Supporting information


**Figure S1** Contraction of hydrogels from different origin over 1 week. From left to right, Rat Tail Collagen, PureCol®, and VitroCol® are shown: the contraction of the gel measured using a Vernier caliper. The results present the average ± SD of the data from three measurements performed on duplicates (*n* = 6).

**Figure S2** Contraction of hydrogels from different origin over 1 week. From left to right, Rat Tail Collagen, PureCol®, and VitroCol® are shown: Each image was segmented in FIJI using Li's Minimum Cross Entropy thresholding method to calculate the area covered by collagen. The results present the average ± SD of the data from three measurements in different xyz positions performed on duplicates (*n* = 6).

**Figure S3** Contraction of hydrogels with and without dermatan sulfate (DS). The contraction of the gel was measured using a Vernier caliper. In gray, the results are shown for gels without the biomolecule. In black, the results are shown for gels made with DS. The results present the average ± SD of the data from three measurements performed on duplicates (*n* = 6).

## Data Availability

The data that support the findings of this study are available from the corresponding author upon reasonable request.
