# Supplementary figures and images for: Evaluation of native and non‐native biomaterials for engineering human skin tissue
Source: Bioeng Transl Med. 2022 Feb 21;7(3):e10297. doi: 10.1002/btm2.10297 (PMC9472026; doi:10.1002/btm2.10297)

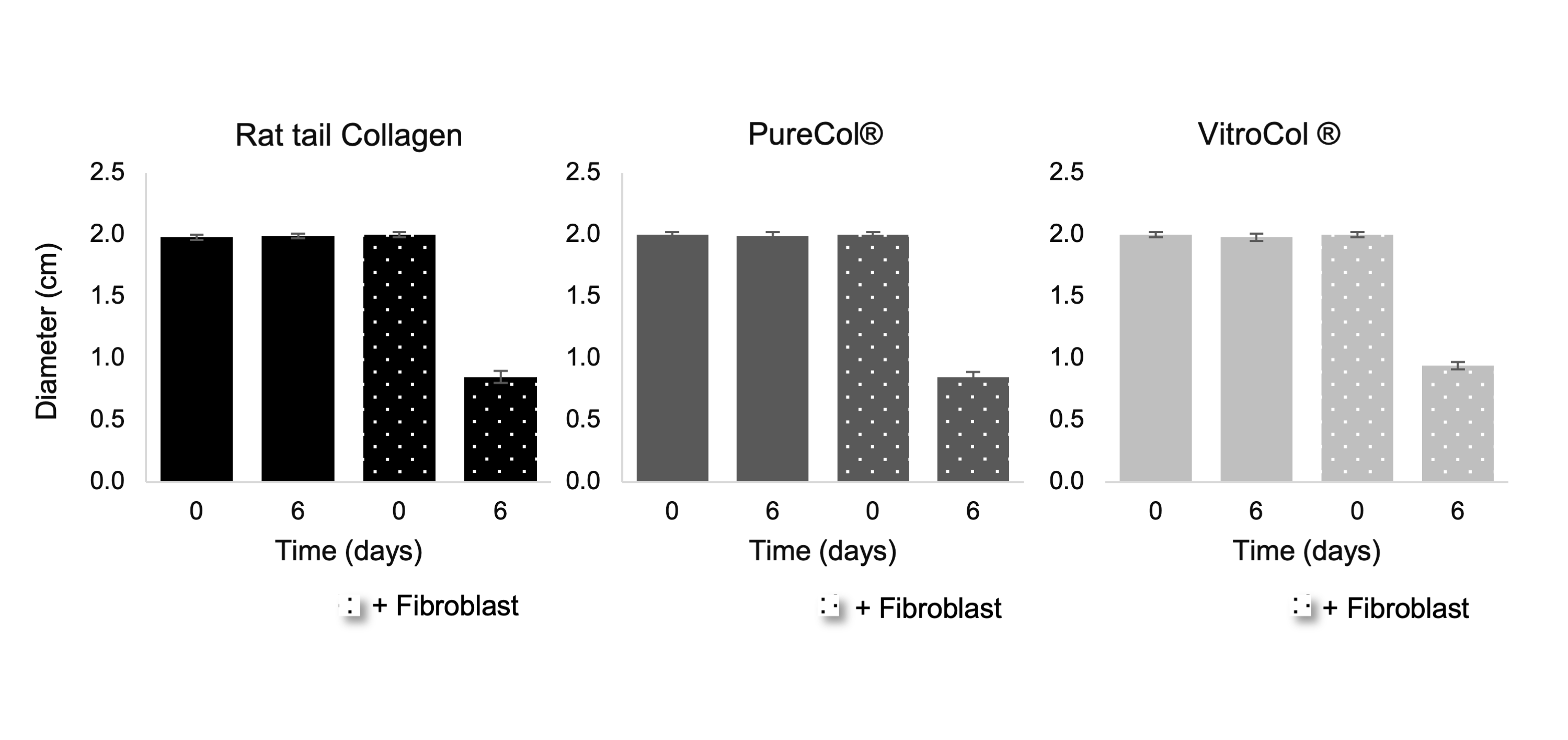

Supplement: Supplementary file 1 — Figure S1 Contraction of hydrogels from different origin over 1 week. From left to right, Rat Tail Collagen, PureCol®, and VitroCol® are shown: the contraction of the gel measured using a Vernier caliper. The results present the average ± SD of the data from three measurements performed on duplicates (n = 6). [file BTM2-7-e10297-s003.tiff]

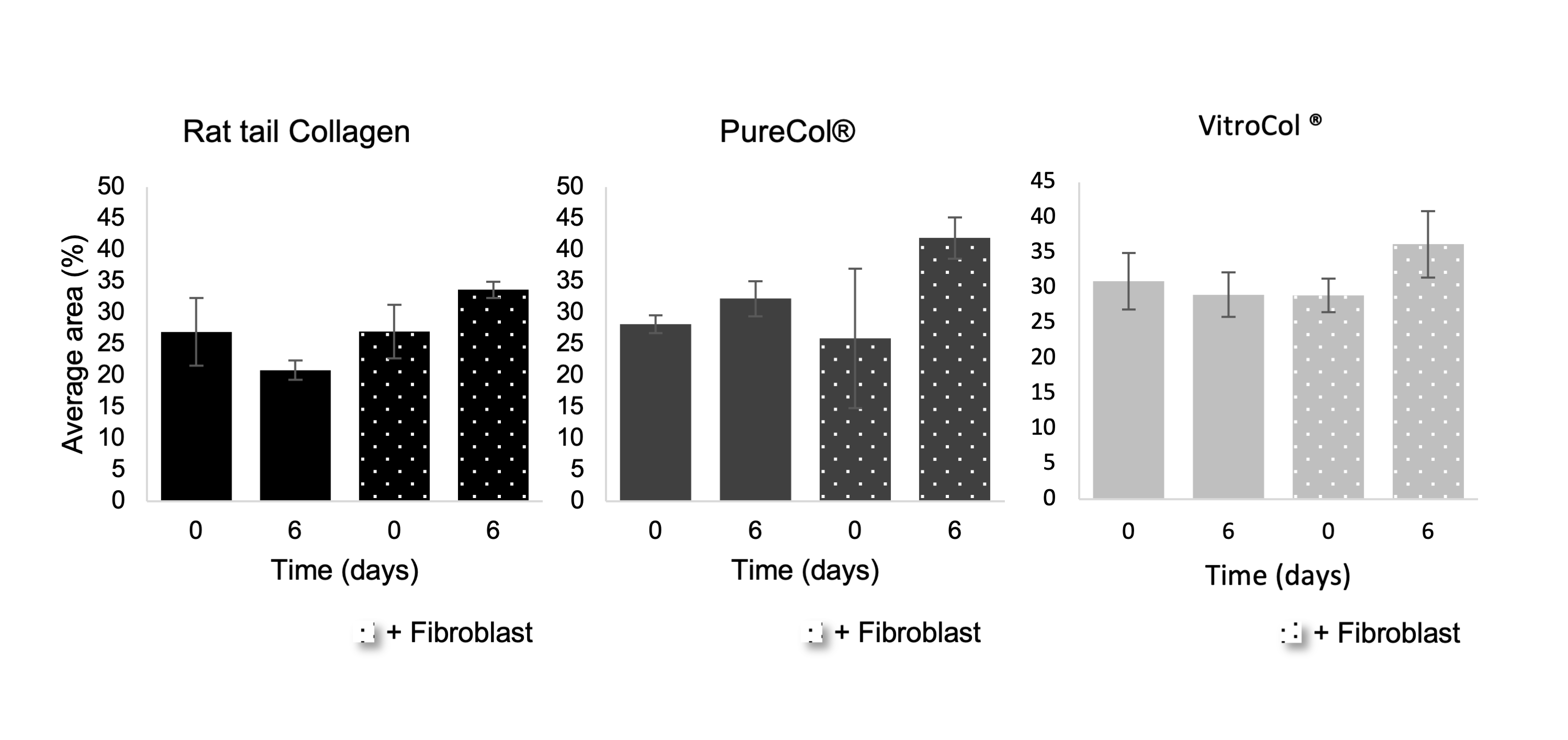

Supplement: Supplementary file 2 — Figure S2 Contraction of hydrogels from different origin over 1 week. From left to right, Rat Tail Collagen, PureCol®, and VitroCol® are shown: Each image was segmented in FIJI using Li's Minimum Cross Entropy thresholding method to calculate the area covered by collagen. The results present the average ± SD of the data from three measurements in different xyz positions performed on duplicates (n = 6). [file BTM2-7-e10297-s002.tiff]

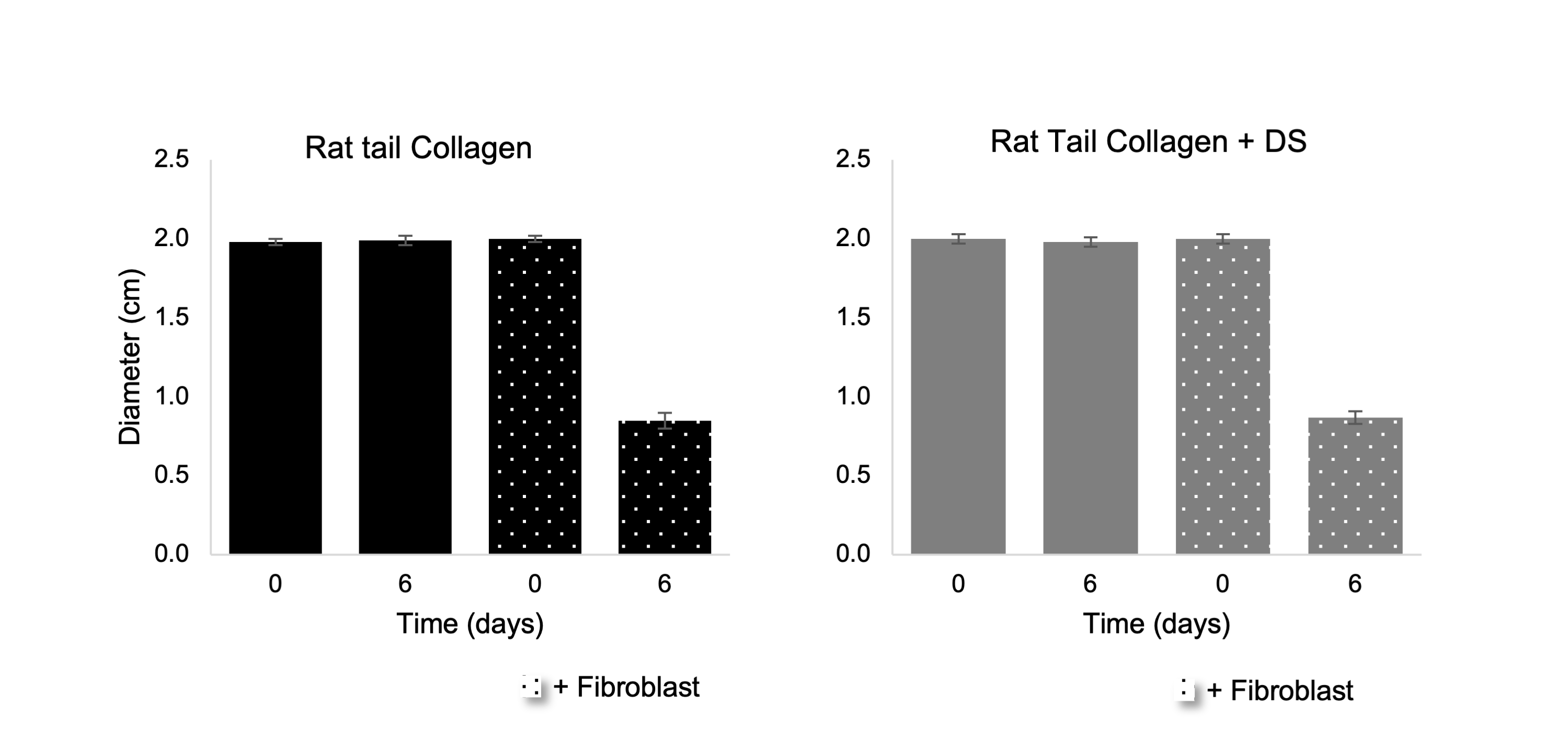

Supplement: Supplementary file 3 — Figure S3 Contraction of hydrogels with and without dermatan sulfate (DS). The contraction of the gel was measured using a Vernier caliper. In gray, the results are shown for gels without the biomolecule. In black, the results are shown for gels made with DS. The results present the average ± SD of the data from three measurements performed on duplicates (n = 6). [file BTM2-7-e10297-s001.tiff]
